# *Chironomus* sp. as a Bioindicator for Assessing Microplastic Contamination and the Heavy Metals Associated with It in the Sediment of Wastewater in Sohag Governorate, Egypt

**DOI:** 10.1007/s11270-023-06179-x

**Published:** 2023-02-24

**Authors:** Azza M. Khdre, Somaia A. Ramadan, Ali Ashry, Mohamed Alaraby

**Affiliations:** Entomology and Environmental Toxicology Group, Zoology Department, Faculty of Science, Sohag University, Sohag, 82524) Egypt

**Keywords:** Microplastic fibers, Wastewater, Sludge, Polyester, *Chironomus*, Chironomid tubes, Heavy metals

## Abstract

The consequences of plastic waste pollution have imposed wide global concerns. One of these consequences is the production of micro- and nanosized particles (MNPLs) from aged plastics. The problem of MNPLs is magnified by their potential to transport various contaminants due to their large surface area and other variable physiochemical properties. From this point on, it is important to know the real concentration of MNPLs in our environment and their potential to internalize wild organisms as well as transfer contaminants that are completely highlighted. As a result, our study is the first to detect MP pollution in the upper Egypt wastewater environment. It could be utilized as a baseline to estimate MP wastes and develop management techniques, particularly in Sohag Governorate. The concentration and characterization of MPs in sludge, water, *Chironomus* sp. larvae, and their tubes were studied in this work. *Chironomus* sp. is a reliable bioindicator prevalent in such contaminated environments, and it was used to demonstrate how MPs invade biological barriers. Our results found that red and blue polyester fibers are much more prevalent than other polymers, colors, and shapes of MPs. While each dry kilogram of wastewater sludge contains 310 ± 84 particles, this amount is reduced to 1.55 ± 0.7 per liter in the water column. Biologically, the present study succeeded in detecting the MPs inside the wild organism, with concentrations reaching 71 ± 21 and 4.41 ± 1.1 particles per gram wet weight in *Chironomus* sp. larvae and their tubes (chironomid tubes), respectively. The potential hazard of MPs stems from their propensity to transfer pollutants. At this point, our findings revealed a corresponding and significant concentration of various heavy metals (Cu, Pb, Cd, and Ni) detected in MPs or *Chironomus* sp. versus sludge. In conclusion, our findings not only proved the presence of MPs in wastewater but also demonstrated their ability to internalize cross-wild organisms, allowing toxins to accumulate inside their bodies, raising concerns about the possible health impacts of plastic pollution.

## Introduction

Microplastics are synthetic pollutants prevalent in both aquatic and terrestrial settings (Malla-Pradhan et al., 2022). During the COVID-19 epidemic, widespread inappropriate use of plastic objects and poor disposal of plastic trash (Silva et al., 2021) exacerbated the plastic pollution catastrophe, necessitating a large and comprehensive response effort (UNEP 2021). Plastic degradation in the environment causes large plastics to reduce into a variety of sizes. Plastics have been classified into different sizes and can be defined as macroplastics (> 25 mm), mesoplastics (5–25 mm), large microplastics (1–5 mm), and small microplastics (1 µm–1 mm) (Gigault et al., 2018; Immerschitt & Martens, 2021). MPs have been recognized in many terrestrial, freshwater, and marine environments in a variety of colors, shapes, sizes, chemical compositions, and concentrations (Campanale et al., 2020). Terrestrial environments have been identified as a source of MPs entering freshwater systems, most likely by runoff from urban, agricultural, and industrial regions, as well as wastewater treatment plant (WWTP) discharge (Anderson et al., 2016). Thus, marine environments have been recorded as the final sink for MP pollution (Nel et al., 2018).

Microplastics are categorized as either primary or secondary based on their origin (Cole et al., 2011). Primary MPs are manufactured in extremely small sizes for specialized applications such as creams, toothpaste, and related products that may enter the freshwater of a faulty WWTP (Wagner & Lambert, 2018). Secondary MPs are large plastics such as fragments and films that are produced when larger plastics are subjected to abiotic effectors such as wind, waves, and ultraviolet radiation (Dawson et al., 2018; Weinstein et al., 2016), as well as biotic stresses such as hydrocarbon-degrading microbes (Eerkes-Medrano et al., 2015). Previous studies on the interactions among both MPs and freshwater biota have shown that MPs can be consumed by freshwater organisms from sediments and that the ability of freshwater invertebrates to take up MPs is dependent on their food habits (Scherer et al., 2017). Macroinvertebrates’ abundance in different ecological niches gives them great importance as bioindicators for assessing MP pollution in the water column and freshwater sediment. In addition to the consumption of MPs by aquatic macroinvertebrates, MPs may be incorporated into larval cases (biological structures) built by several epibenthic insects, such as caddis fly (*Trichoptera*) species in freshwater environments (Ehlers et al., 2019). Therefore, larval cases built by aquatic insects may play a role in MPs’ assessments of freshwater systems and, consequently, as bioindicators for MPs.

Nevertheless, while MPs are relatively inert, they can adsorb toxins such as organic pollutants and heavy metals due to their unique characteristics (e.g., hydrophobicity), which vary according to the different polymers (Collicut et al., 2019; Guo & Wang, 2019). The ability of MPs to adsorb increases with decreasing size (Wagner et al., 2014), and the quantities of organic pollutants adsorbed by MPs may become 1 million times higher than the ambient concentration (Mao et al., 2021). Therefore, MPs could be a source of highly toxic substances when ingested by aquatic organisms (Koelmans et al., 2014; Wang et al., 2017). For example, heavy metals and pathogenic microbes can be conveyed to humans through the physical porous structure of microplastics, which allows harmful heavy metals and microbes to adhere to the surface and be passed via aquatic bodies (Abuwatfa et al., 2021; Chowdhury et al., 2021).

Furthermore, higher trophic level animals are more vulnerable to MP toxicity because MPs tend to stay in their bodies for longer periods (Wagner & Lambert, 2018). Previous research found plastic pollution in marine environments. However, there have been few records of MPs in freshwater environments. These reports have mainly focused on the biota occupying the higher aquatic food webs, such as fish (Foekema et al., 2013; Horton et al., 2018). Recent field data have highlighted MP ingestion by freshwater invertebrates (Setälä et al., 2016; Scherer et al., 2017; Bour et al., 2018; Sfriso et al., 2020; Pan et al., 2021). More recently, Bertoli et al. (2022) focused on the influence of feeding guilds and habits of invertebrates on MP ingestion. Furthermore, interactions among both MPs and contaminants such as heavy metals have received much interest (Brennecke et al., 2016; Wang et al., 2017; Wijesekara et al., 2018). Despite the wide variety of aquatic insects and their ecological relevance (Suter & Cormier, 2015), few recent studies have focused on the consumption of MPs by freshwater insects (Kim et al., 2018; Nel et al., 2018; Windsor et al., 2019). In the Sohag Governorate, no data is available on the occurrence of MPs in sediment, water, or freshwater insects. One of the most polluted wastewater basins in the Sohag Governorate was selected for this study. A large amount of effluent is discharged into this basin daily, and the water is used to irrigate land utilized for agriculture. Furthermore, wastewater sludge can be utilized as soil fertilizer, causing MPs to be transferred to the surface of agricultural land, which is a major issue. As a result, the current study aims to (i) quantify the concentration of MPs in the current wastewater basin's sludge and water; (ii) determine whether MPs are present in the bodies of the most abundant insect larvae (*Chironomus* sp.); (iii) determine whether the larval tubes of *Chironomus* sp. may be employed as a bioindicator of MPs; and (iv) determine whether the MPs act as metal carriers in freshwater.

## Materials and Methods

### Study Area

**Fig. 1:**
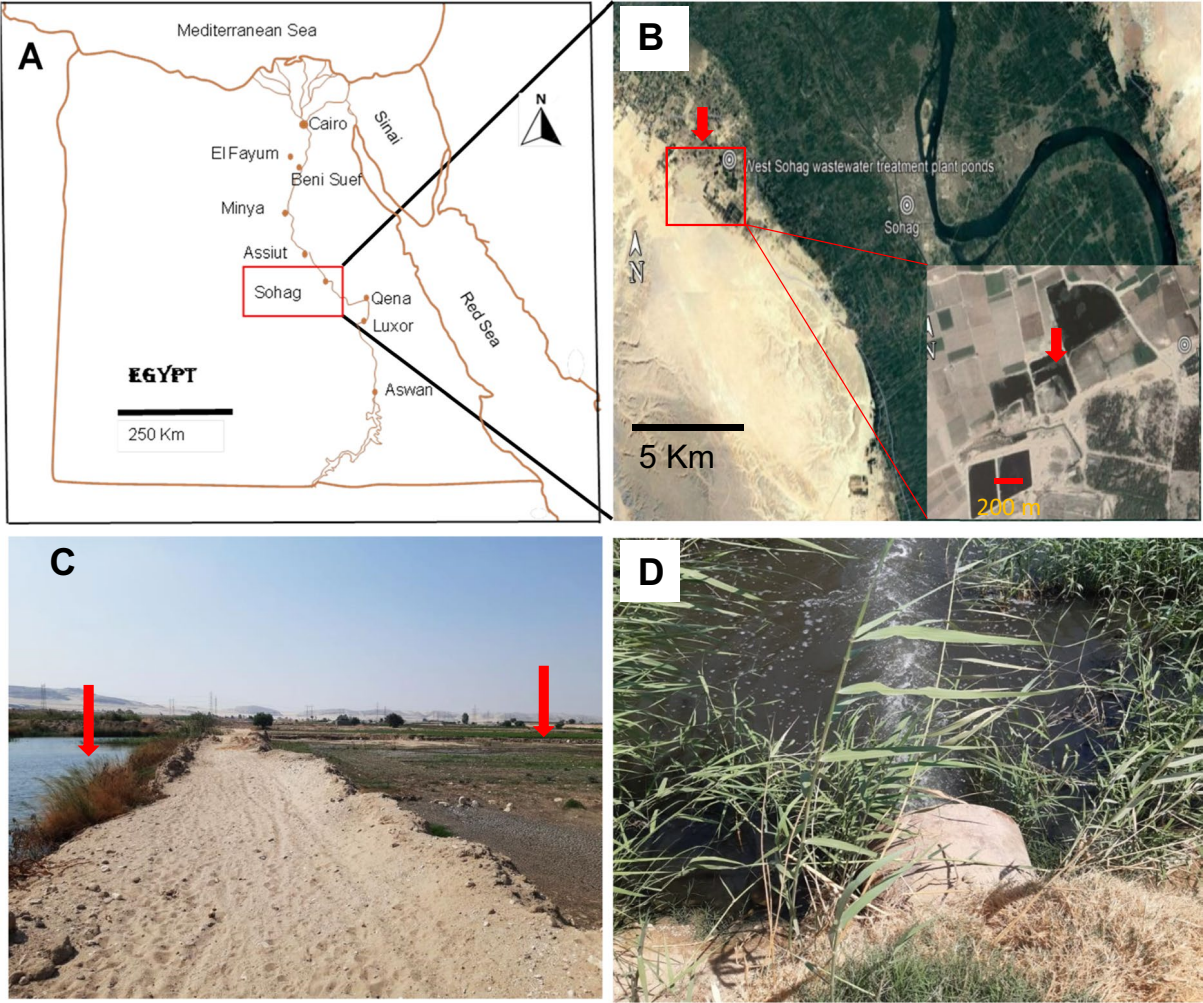
Egypt map showing Sohag Governorate (**A**). Google Earth photo showing collecting site (**B**). Photographs showing the location of the sampling site within the wastewater basin (**C** and **D**)

The collection basin is in a desert region 10 km west of Sohag Governorate (26° 33′ 47″ N and 31° 36′ 11″ E) (Fig. 1). The basin is rectangular, covering around 3.786 km^2^, and has an average depth of 1 m. The basin is subjected to various pollution sources, such as the continual discharge of wastewater from a nearby wastewater treatment plant, human activities because of agriculture development, animal husbandry, and sewage discharge from nearby communities. The basin is distinguished by the existence of large quantities of algae, and the water flow is so slow that it can be considered stagnant water. Patches of different plants were observed at the edges of the basin (Fig. 1).

### Sample Collection

#### Water Collection

Samples of water were taken along the basin’s edge in 2021. Triplicate samples were taken at five separate horizontal points (p1, p2, p3, p4, p5) of the surface water at a depth of about 0–20 cm to obtain a comprehensive distribution pattern of MPs present in the basin. A total of 75 l of water were collected using a steel bucket and stored in clean glass containers for laboratory analysis.

#### Sludge Collection

With a stainless-steel spoon, 15 sludge samples (each sample weighing 2 kg) were taken from the upper sludge (0–5-cm depth). After that, the sludge was deposited in closed buckets for laboratory analysis.

#### *Chironomus* sp. Collection

A 0.5-mm mesh size net was used to collect *Chironomus* sp. larvae from five different points. The collected larvae were immediately fixed in 70% alcohol in 100-mL screw-capped glass vials to prevent ejection of gut contents, which could change MP estimation, as reported by Nel et al., (2018), and the other group was immediately immersed in an ice box to determine concentrations of heavy metals and stored in the lab’s deep freezer*.* The chironomid tubes were collected from sediment samples and preserved in a deep freezer to extract MPs from them.

### Laboratory Analysis

#### Sludge Characteristics

Samples of sludge were weighed and dried at 60 °C for 3 days. The particle size and organic content of previously dried sludge were determined using 15 subsamples. Sludge fractions were separated using a mechanical sieving system comprised of standard sieves with varying pore sizes (4000 µm, 2000 µm, 500 µm, 212 µm, and 53 µm). Each sludge fraction was collected with great care and weighed to calculate its percentage.

The organic content of dry powdered sludge was determined using 15 replicates (2 g each). The samples were put in pre-weighed porcelain crucibles and incinerated at 600 °C for 6 h; then, the weight of organic material was calculated from the following equation: (loss of burning weight)/(weight of dry sludge) = 100% organic matter.

#### Water Microplastic Extraction

Samples of the collected water (1 l) were transferred into glass beakers. Then, 20 ml of 35% hydrogen peroxide (H_2_O_2_) was added overnight (Hidalgo-Ruz et al., 2012) to digest the majority of the organic debris without impacting any plastic material (Dahms et al., 2020). The digested samples of water were filtered via filter paper made of cellulose nitrate with a 0.45-µm pore size (Ribeiro-Brasil et al., 2020) using a vacuum filtration system (the filter is checked under a microscope before being used to confirm that it is free of contaminants). The MP particles on the filter paper were allowed to dry at room temperature in covered glass Petri dishes before being examined further.

#### Microplastic Extraction from Sludge

The samples of sludge were put in clean glass containers and then dried in an oven at 60 °C for 48 h. After drying, each 100-g sludge sample was placed in a clean 1-l beaker. A density separation method was carried out to separate MPs from denser natural particles. A freshly prepared hypersaline solution of NaCl/NaI (0.5 g/cm^3^) was added to the sludge samples until the beaker contents reached about 500 ml (Coppock et al., 2017). The beakers were covered with aluminum foil and at 200 rpm shaken for 2 days on a shaker table (OS-2000, open-air–dual-action shaker, JEIOTECH, Korea) to separate any MPs. The floating supernatants were transferred to a second beaker and left for 24 h to settle any sludge and allow the MPs to float. To gather all MP particles, the supernatant was then filtered through a 0.45-µm filter and preserved for microscopic identification. To guarantee that all the MP had been extracted from the sludge, all the previous operations were repeated several times.

#### Extraction of Microplastics from *Chironomus* sp. Larvae and Their Tubes

The fourth instar of *Chironomu*s sp. larvae was carefully rinsed in ultrapure water to prevent any exterior contaminants and was then separated into 15 groups (20 individuals each). An analytical balance was used to determine the weight of each group. Each group’s larvae were placed in glass beakers, and 20 ml of H_2_0_2_ (35% V/V) was added and microwaved for 1 min to digest organic matter. The beakers were closed with aluminum foil and shaken for 48 h at 200 rpm to completely break down the larval tissues (Windsor et al., 2019). The supernatant was vacuum filtered through filter paper (0.45 µm) and dried in an oven at room temperature in covered Petri dishes. The extracted MPs were stored for further analysis*. Chironomus* sp. tubes were also processed according to the steps described above using 15 samples (each sample containing 10 tubes).

#### Microplastic Identification and Characterization

All stored MPs were recognized visually using a dissecting microscope equipped with a digital camera (Carl Zeiss Suzhou Co.). A heated needle was further applied to confirm MP identification (Windsor et al., 2019). The shapes and colors of MP particles were also detected and photographed. Using the Image J program, the length and diameter of each MP were measured. An attenuated total reflection Fourier transform infrared spectroscopy (ATR-FTIR, Alpha Bruker platinum, 1–211-6353), using zinc slender crystal with an incident angle of 45 ± 15 and 560 scan time (24 s) with a resolution of 4 cm^−1^ used to validate MPs identification (range: 4000–400 cm^−1^). MP particles of various colors and shapes were chosen for investigation. The data obtained was manipulated with the OPUS software (Bruker Optics GmbH). The type of polymer present was detected by comparing the obtained spectra with the reference spectra reported by Primpke et al., (2018).

#### Heavy Metal Analysis

The levels of the four heavy metals (Cd, Ni, Pb, and Cu) were measured in sludge samples, MPs that were isolated from the sludge, and *Chironomus* sp. larvae bodies.

Fifteen samples of sludge were subjected to acid digestion following the method of Islam et al., (2019) with some modifications. In a Teflon vessel, a 0.5-g dried powdered sludge sample was mixed with 5 ml (60%) HNO_3_ and 2 ml (50%) H_2_O_2_ and microwaved. The digested sludge was filtered and kept in 50-ml polypropylene tubes. The metal concentration was estimated using ICP/OES (ALBHA Standard Edition-23rd). Throughout the examination of the sediment samples, blank reagents with no additional samples of sediment were also verified, and the investigated metals were undetectable.

Fifteen replications Samples of dried MP particles (≈ 12 mg each) extracted from sludge were digested according to the Ashton et al., (2010) method. A modified aqua regia extraction was used to remove metals from MPs. Aqua regia was prepared by adding 3 M HNO_3_ and 2 M HCl in a 1:3 ratio. Microplastic particles were placed in clean, screw-capped tubes, which were then filled with 15 ml of 20% aqua regia. Clean, screw-capped tubes were shaken for 24 h at 200 revolutions per minute at room temperature. A volume preparation of 20% aqua regia was also employed throughout the extraction for all blanks and calibration to guarantee precision in the extracted analysis. Subsequently, these digests were transferred to clean Teflon tubes and analyzed to evaluate Cd, Ni, Pb, and Cu concentrations.

To assess the presence of heavy metals in the larvae of *Chironomus* sp., fifteen samples of larvae (10 individuals each) were pressed in distilled water, and then they were dried at 60 °C for 3 h in crucibles. The samples were processed in 5 ml of concentrated HNO_3_ at 80 °C, and the volume was increased to 10 ml of distilled water (Timmermans et al., 1992). Then, ICP/OES was used to calculate the Ni, Pd, Cd, and Cu concentrations. Plank samples were utilized in the absence of larvae.

### Precautions

Special attention was taken to restrict sample contamination. MP extraction was carried out in a clean area, and all tools utilized were cleaned with distilled water regularly. A covered glass Petri dish was used to store samples. As a negative control, sample blanks and airborne controls were employed. During this study, a total of five sample blanks were generated. The NaCl/NaI solution used in the density separator was filtered before being used for the sediment sample blanks. The stereomicroscope was then used to examine the filter. During the visual observation procedure, three opened Petri plates filled with distilled water were placed close to the microscope as airborne controls. Controls were inserted 15 min before the sample observation and removed 15 min after the visual examination by the MPs was completed. The controls were then visually examined using the same approach as the samples.

### Statistical Analysis

Descriptive statistics (mean and standard deviation SD) were used to describe the basic features of MP concentration in wastewater, sludge, larvae of *Chironomus* sp., and chironomid tubes in the basin. One-way ANOVA, *χ*^2^ test, and the paired *t*-test were used to determine significant differences in MP-related data among groups using the IBM SPSS 21.0 software. A statistically significant difference was accepted when *p* ≤ 0.05 was used (Wong et al., 2020, 2021).

## Results and Discussion

### Microplastic Abundance in the Wastewater Basin

All the water and sludge samples contained microplastics. The average abundance of MPs in sludge was 310 ± 84 particles kg^−1^ dry weight. The average abundance of MPs in water, on the other hand, was 1.55 ± 0.7 particles l^−1^. The MP level was relatively high in the sludge. This might be attributed to the fine sludge shape (sewage sludge) that was observed in the present basin, which has high organic content (27 ± 4), which in turn may allow the sludge to bind with MPs and become a trap for them (Anderson et al., 2016; Teuten et al., 2009). Other evidence for the relatively high level of MPs in sludge might be provided by some of the basin characteristics, such as very slow water flow to the extent that it can be considered stagnant water. This result supports the finding of Nel & Froneman (2015), who reported that the highest MP abundance in sediment was recorded with reduced river flow, which represents one of the most important environmental factors influencing the MP distribution in the aquatic system. With the decrease in water velocity, the installation of MPs increases due to the adsorption of natural chemicals, biofouling, and gravity (Mao et al., 2020). As a result, the low abundance of MPs in the surface water of this basin might be explained by the fact that MPs associated with sludge can't be easily resuspended in the surface water (Mao et al., 2020), because the binding of MPs might require more energy to separate them (Dikareva & Simon 2019; Dahms et al., 2020). Furthermore, the closed environment of the present basin, where a point source of MPs is present, could increase the abundance of MPs gradually through long-term degradation and accumulation (Eerkes-Medrano et al., 2015). Compared to previous studies, Dahms et al., (2020) reported that the mean number of MPs throughout an urban African stream was 166.8 particles kg^−1^ d.w. Also, data reported by Redondo-Hasselerharm et al., (2018) revealed that the Rhine River had the highest MP concentration of all freshwater systems studied (4000 particles kg^−1^ d.w.). The variation in MP concentrations between freshwater systems is likely to be significant. In general, the abundance of MPs is strongly related to the sampling locations and is dependent on urbanization and population density, with anthropogenic activities serving as a major source of MP contamination (Kataoka et al., 2019; Naqash et al., 2020; Zhang et al., 2019).

The presence of MPs in sludge may allow benthic macroinvertebrates to ingest them, thereby reflecting the MP loads in their bodies. *Chironomus* sp. larvae can be considered an ideal organism for assessing MP pollution in different aquatic habitats (Bere et al., 2016; Nhiwatiwa et al., 2017). The present results confirmed the role of *Chironomus* sp. larvae in determining MP pollution in freshwater, where the average MP load in *Chironomus* sp. larvae was 71 ± 21 g^−1^ w.w. For comparison, previous studies (Dahms et al., 2020) on *Chironomus* sp. larvae obtained from the freshwater of the African river of Johannesburg reported that the ingesting of MP was lower (56.2 particles g^−1^ w.w.) than that detected in the present study. However, the abundance of MPs detected in *Chironomus* sp. larvae of the Ogun River in Nigeria was very high (291 ± 26 g^−1^ w.w.) (Akindele et al., 2020). These results indicate that the difference in MP concentrations in deposit feeders (*Chironomus* larvae) may be related to the different plastic pollution levels in the various freshwater habitats.

Based on the wet body weight of the fourth instar larvae, the weight of different samples ranged from 120 to 180 mg, which was significantly different (*t* = 15.78, *p* = 0.001). Also, statistical analysis indicated a significant difference between these samples regarding MP load (*t* = 6.9, *p* = 0.02). However, the correlation between sample weights and the number of MPs ingested by larvae after the standardization of data was relatively weak (*r* = 0.275, *p* < 0.05). Microplastic sample load variation was possibly due to an unequal distribution of MPs on the sludge surface, which could have had an influence on the uptake of MPs by the larvae. The standardized data showed that there was a weak correlation between sample wet weight and the number of MPs burned by larvae. This indicates that there's a limited relationship between larval size (weight) and the ingestion of MPs.

Chironomid larvae have been observed as tube-building larvae of some Diptera in freshwater (Chaloner & Wotton, 1996). The MPs incorporated into the *Chironomus* sp. tubes averaged 4.4 ± 1.1 particles g^−1^ w.w. The results showed that *Chironomus* sp. larval tubes have an important role as freshwater MP bioindicators. Ehlers et al., (2019) discovered that freshwater caddisfly larvae incorporated MPs into their biological cases, and marine polychaete species fixed MPs in their tubes (Nel & Froneman, 2018). According to Ehlers et al., (2019), the MP fixation process occurred across different ecosystems, and the analysis of the tubes gave information about MP ecosystem characteristics. Therefore, MP loads in *Chironomus* sp. and their tubes in different aquatic systems (differing in plastic pollution) still need further study in the future.

### Characteristics of MPs in the Basin

**Fig. 2:**
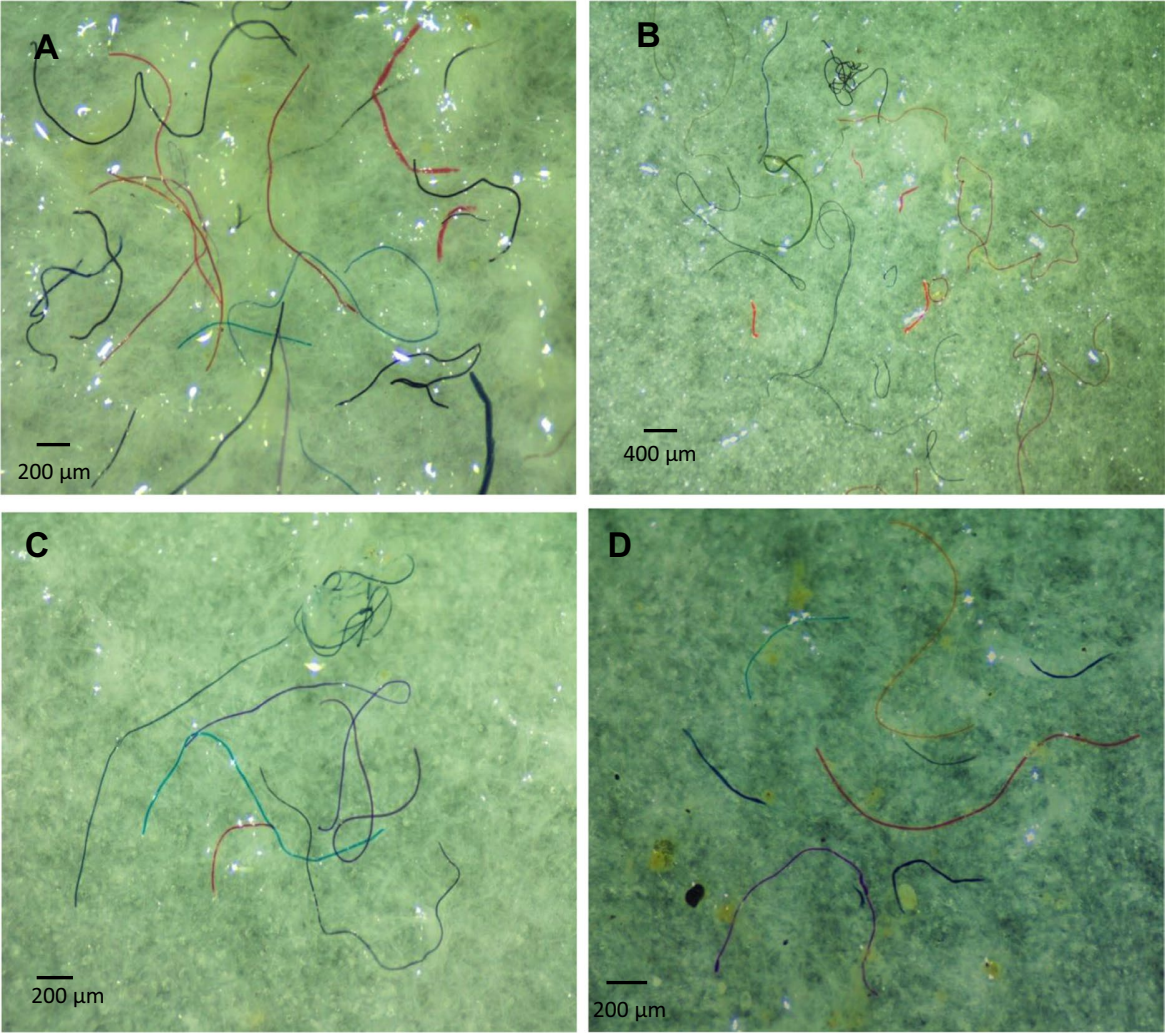
Photographs of microfibers were collected from sludge, water, *Chironomus* sp., and chironomid tubes (**A**, **B**, **C**, and **D** respectively)

The characteristics of MP particles were similar among different samples of water, sludge, and *Chironomus* sp. Larvae and their tubes. According to their shape, fibers were the only MP found in all samples, comprising 100% of the total identified particles (Fig. 2). The dominance of fibers in all the samples collected suggests that the source of MPs in the basin is more related to the direct primary inputs that originate from the large amounts of sewage effluents from WWTP, where textile-derived fibers are dominant. Moreover, semi-urban areas around the present basin can contribute to a higher abundance of MP fibers as the wastewater originating from washing clothes and plastic carpets are discharged directly into the basin without treatment. Browne et al., (2011) reported that sewage polluted by fibers from washing clothes can produce 100 fibers/l of effluent. Napper & Thompson, (2016) reported that domestic sewage from washing machines contains large quantities of artificial fibers. In general, urban effluent, particularly domestic pollution, is a major contributor to the abundance of microfibers in aquatic systems (Luo et al., 2019). Furthermore, according to Cole et al., (2011) and Horton et al., (2017), agriculture was the primary human activity in the current area, capable of producing a significant number of plastic fibers. The present results are like other previous field freshwater studies in China in which the fiber MP was the most abundant in both water and sludge, being 48% and 84%, respectively, in the Tiahul lake (Su et al., 2016); 41.9% and 91.9%, respectively, in the Dongting lake; and 44.2% and 83.9%, respectively, in the Hong Lake (Wang et al., 2018). Also, studies in Germany on treated wastewater carried out by Mintenig et al., (2017) revealed that synthetic fibers dominated in more than 80% of the samples. Likewise, fibers have been recorded as the most frequent type of MP in aquatic macroinvertebrates, accounting for more than 50% of the total MPs in the mollusks obtained from the northern part of the Persian Gulf (Naji et al., 2017). Also, 87% of MPs ingested by freshwater annelids (*Tubelix tubelix)* were microfibers (Hurley et al., 2017). Akindele et al., (2019) reported that 100% of the MPs ingested by two species of gastropods in the Osun River, Nigeria, were fibers.

**Fig. 3:**
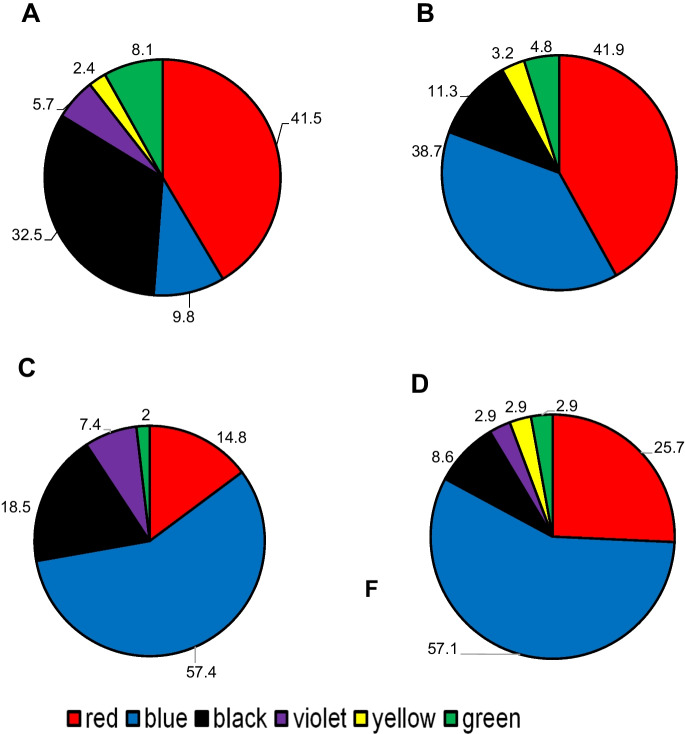
Relative percentages of different colored MPs were found in samples collected from sludge (**A**), water (**B**), *Chironomus* sp.(**C**), and chironomid tubes (**D**)

The distribution patterns of MP colors in sludge, water, *Chironomus* sp. larvae, and their tubes are presented in Fig. 3A–D. Microfibers were found in a wide spectrum of colors, including blue, red, black, green, yellow, and violet. Both red and blue were the dominant colors in all samples of MPs extracted from sludge, water, and *Chironomus* sp. larvae, and their tubes, which comprised 51.3%, 80.6%, 72.2%, and 82.8%, respectively. In addition, the proportion of black color detected was higher in sludge (32.5%) than in water, *Chironomus* sp. larvae, and their tubes. Significant differences were observed among the color proportions in the samples of sludge, water, *Chironomus* sp. larvae, and their tubes (*χ*^2^ = 67.04, *p* < 0.0001). Moreover, differences between blue color percentages in sludge and both *Chironomus* larvae and their tubes were also significant (*χ*^2^ = 33.2, *p* < 0.0001). Six different colors were observed, with significant percentage differences among them. Similar results of a wide spectrum of colored MPs have been detected in samples of water, sludge, and macroinvertebrates in previous research (Zhao et al., 2015). The wide range of colors is most likely due to the abundance of colored plastics that are widely produced and consumed. Regarding MPs’ colors, blue is the most abundant color in both samples of *Chironomus* sp. larvae and their tubes (approximately 57% and 49%, respectively). As the *Chironomus* sp. larvae feed on and construct their tubes from materials available in the sludge where they inhabit, this may suggest the preference of larvae for this color, which may be like their food. Shaw & Day, (1994) reported that aquatic biota may feed on MPs that resemble their prey in color and shape. Furthermore, the larvae’s choice of blue for their tubes might indicate that they selected the color to blend in with their surroundings. As a result, the low proportion (10.8%) of this color in the sludge, where *Chironomus* sp. larvae were the only species found in the analyzed basin sludge, might be explained.

**Fig. 4:**
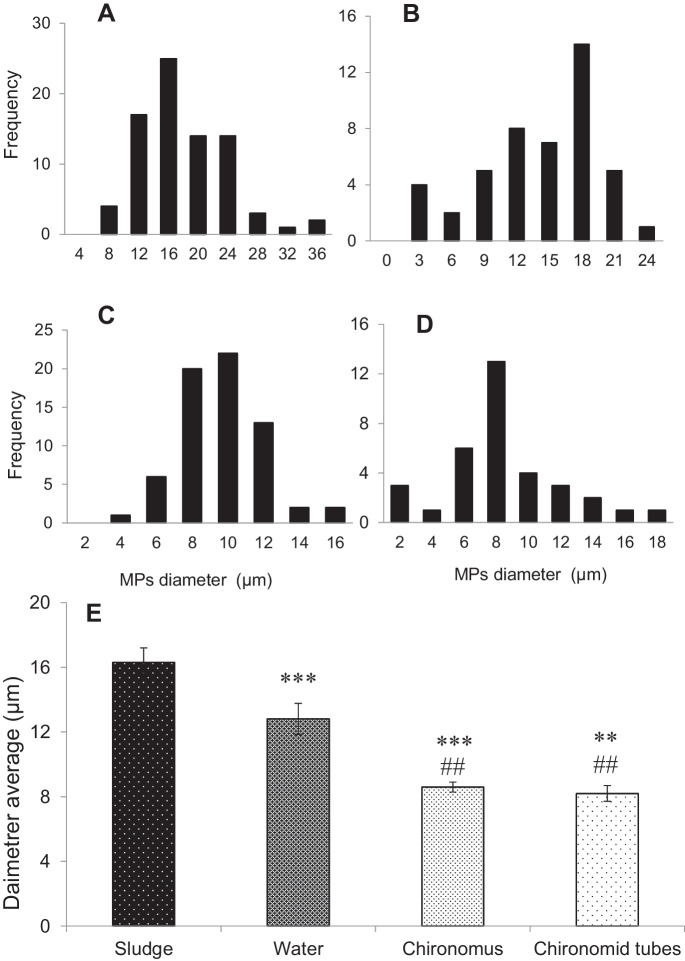
Diameter frequency of fibers collected from sludge, water, *Chironomus* sp., and chironomid tubes (**A**, **B**, **C**, and **D**, respectively). The average diameter is 16.3 ± 6.1 µm, 12.8 ± 5.8 µm, 8.6 ± 2.3 µm, and 8.2 ± 3.6 µm (**E**). ^##^ and **p < 0.01, ***p < 0.001. *For comparing vs sludge, ^#^for comparing vs water

**Fig. 5:**
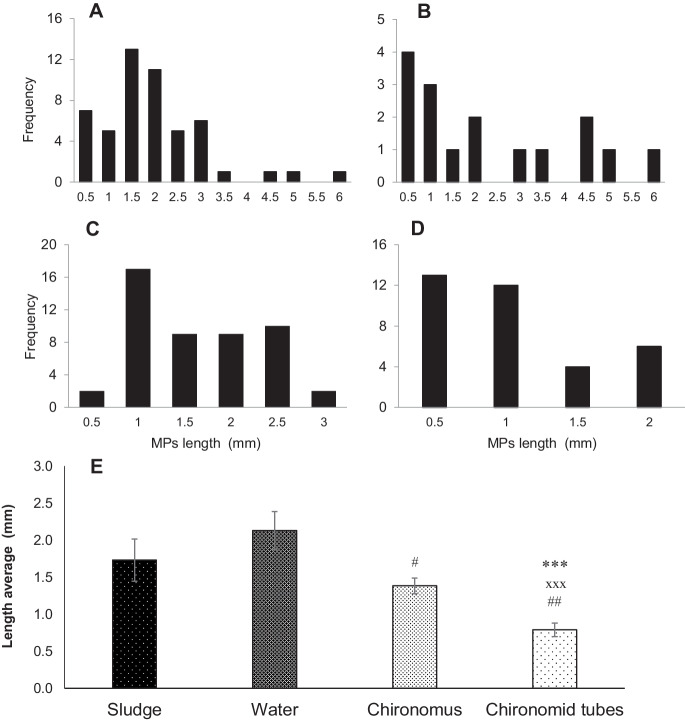
Length frequency of MPs collected from sludge, water, *Chironomus* sp., and chironomid tubes (**A**, **B**, **C**, and **D**, respectively). The average length is 1.7 ± 1.1 mm, 2.1 ± 1.8 mm, 1.4 ± 0.7 mm, and 0.8 ± 0.5 mm (E). ^#^*p* < 0.05, ^##^*p* < 0.01 and ***, ^xxx^*p* < 0.001, *vs sludge, ^#^vs water, and ^x^vs *Chironomus*

The size-frequency distribution of microfibers observed in the different samples is presented in Fig. 4A–E. Within the diameter range of 2–36 µm detected in the different samples, the highest mean diameter was observed in the fibers extracted from sludge (16.3 ± 6.11 µm) followed by those detected in water (12.8 ± 5.8 µm) Fig. 4 A and B. Microfibers in the diameter range of 8–10 µm represented the highest frequency of diameter in the sample of *Chironomus* sp. larvae, accounting for the highest proportion of 65% of the detected fibers (Fig. 4C). The smaller fiber diameter range of 6–8 µm was more frequently observed in *Chironomus* sp. tubes, accounting for 57% of all microfibers detected in tubes (Fig. 4D). Regarding microfiber diameter distribution, there were significant differences between sludge fibers and detected in *Chironomus* sp. larvae and their tubes (*p* < 0.001) (Fig. 4E). The trend of microfiber length distribution in the different samples was somewhat like that of microfiber diameters, where the highest lengths were detected in the samples of sludge and water Fig. 5A–E. In sludge samples, the length range of 0.5–2 mm was the most common, accounting for 47% of detected fibers with an average of 1.7 ± 0.3 mm, while in water the average length was 2.1 ± 1.8 mm (Fig. 5 A and B). However, the smaller fibers were the more frequent fractions in both *Chironomus* sp. larvae and their tubes, where the mean lengths were 1.4 ± 0.8 mm and 0.8 ± 0.5 mm in Fig. 5 C and D respectively. Regarding microfiber lengths, there was no significant difference between sludge and *Chironomus* sp. larvae. Meanwhile, the mean length of microfibers in *Chironomus* sp. tubes was significantly shorter than those in sludge, water, and *Chironomus* sp. larvae (*p* < 0.001) Fig. 5E. According to the previous research's categorization of plastic size (Gigault et al., 2018), the MP size identified in the current study is described as “small microplastic.” The tiny size of MPs extracted from sludge may explain the high abundance of MPs within *Chironomus* bodies since the fine size is regarded as a critical feature in allowing a chance for ingestion, which increases MP bioaccumulation in diverse organisms across food webs (Bagheri et al., 2020; Naqash et al., 2020). Moreover, previous research has found a significant abundance of bacteria associated with MPs originating from WWTP effluent (McCormick et al., 2014). This might be an important nutrient source for aquatic biota (Hurley et al., 2017). Furthermore, aquatic macroinvertebrates have been demonstrated to preferentially consume microbe-colonized debris (Chung & Suberkropp, 2009). Preliminary visual examination indicated the presence of organic materials on certain MP fibers, suggesting that tiny fibers might be inadvertently consumed by *Chironomus* sp. larvae.

Most MP sizes extracted from *Chironomus* sp. larvae were significantly smaller in diameter than those extracted from both sludge and water. This result may be explained in two ways: the first is that the *Chironomus* sp*.* larvae have selective feeding on small MP fibers, and the second is that a reduction in MP size may occur inside their gut because of biodegradation. This result confirms the finding of Yang et al., (2019), who reported that some bacterial strains, such as *Bacillus* sp. and *Enterobacter alsuriae* degraded polyethylene MPs isolated from the gut of a waxworm (*Plodia interpunctella*). Different from what was extracted from water and sludge, most of the fibrous MPs extracted from *Chironomus* tubes were significantly smaller either in diameter (*p* < 0.01) or length (*p* < 0.001). This reduction might be attributed to the high selectivity of *Chironomus* sp. larvae in the uptake of MPs for constructing their tubes. Additionally, a further reduction in the MP fiber length was recorded in the *Chironomus* tubes when compared to the MP fiber length extracted from the *Chironomus* themselves. This suggests the smaller fibers may be easier to extract through the digestive system of the larvae and accumulate in the tubes.

**Fig. 6:**
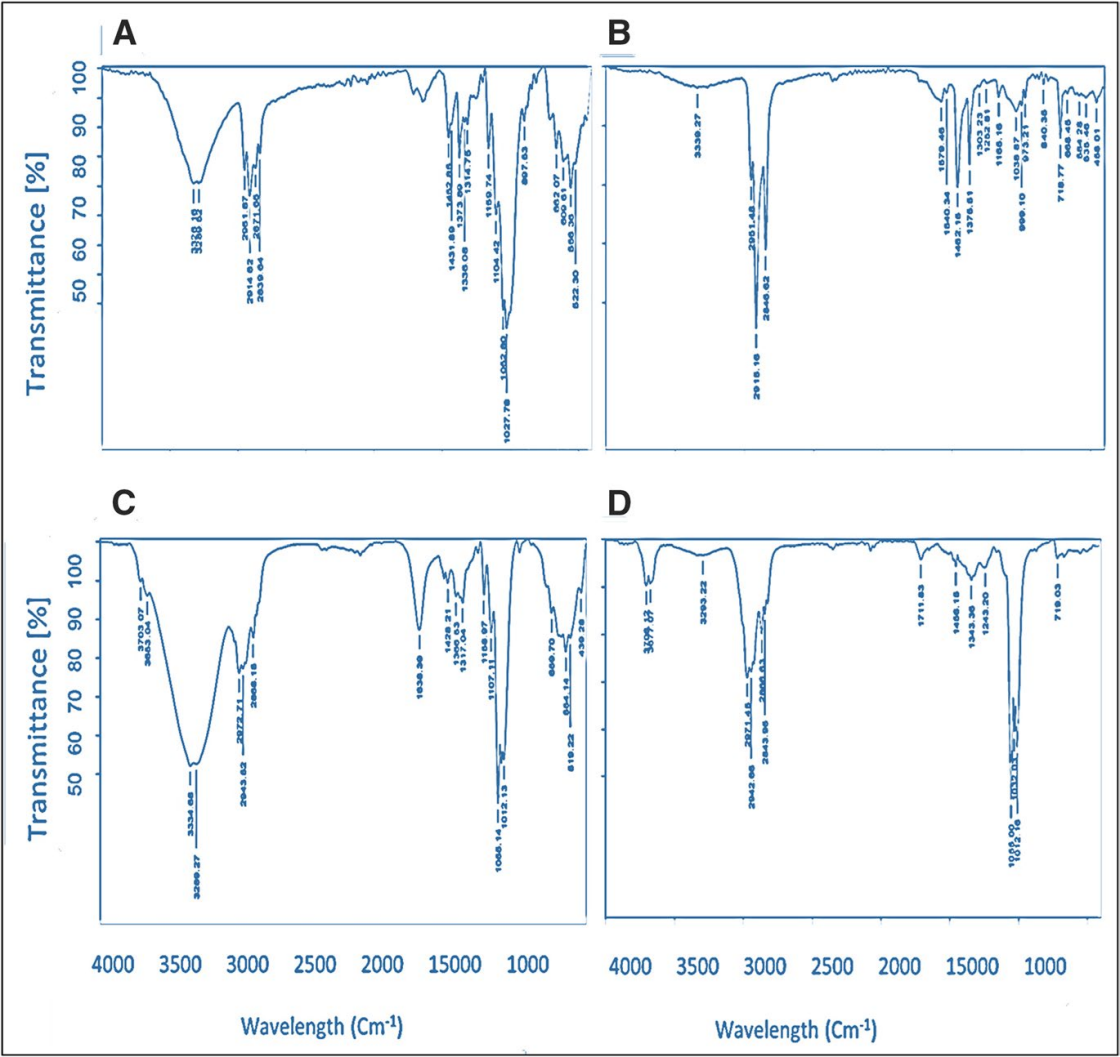
Fourier transform infrared spectroscopy (FTIR) spectra of MPs were collected from sludge, water, *Chironomus* sp., and chironomid tubes (**A**, **B**, **C**, and **D**, respectively)

According to the chemical composition of microfiber, polyester was the only polymer type identified among the randomly selected fibers from sludge, water, and *Chironomus* sp. larvae, and their tubes in Fig. 6A–D. Household wastewater, which is discharged into the basin, may be the main source of polyester particles since polyester is widely used in textiles such as clothes and ropes.

### Metal Accumulation by Microfibers, Sludge, and *Chironomus* sp. Larvae

**Fig. 7:**
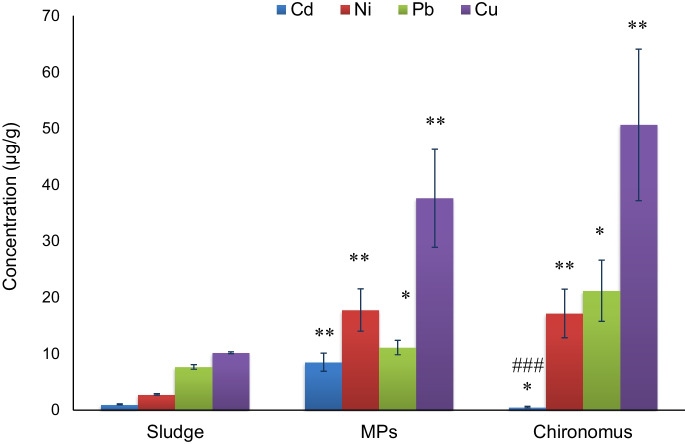
The concentration of heavy metals. *p < 0.05, **p < 0.01 and ^###^*p* < 0.001. *vs sludge, ^#^vs MPs

Heavy metal concentrations in the sludge, *Chironomus* sp. larvae, and MPs are presented in Fig. 7. MPs contained a high concentration of heavy metals on their surface (9–38 μg/g) as compared to sludge samples (1–10 μg/g) indicating their role as a potential vector in transport. The following equation (Selvam et al., 2021) was used to calculate the partition coefficient of heavy metals on MPs’ surfaces from the sediment:

MeP/MeS = Kmps

The metal concentration on the MPs’ surfaces is shown by MeP, while the metal content in the sludge is indicated by MeS. Furthermore, Cd and Ni had higher Kmps values than Cu and Pb.

The heavy metal concentration in *Chironomus* sp. was estimated and determined to be (0.8 to 51 ug/g). Heavy metal concentrations in *Chironomus* sp. larvae were found to be in the following order: Cu > Pb > Ni > Cd (Fig. 7). Metal content in *Chironomus* sp. larvae, MPs, and sludge was analyzed, and it was revealed that heavy metal content in *Chironomus* sp. was more closely related to the metal content of MPs isolated from sludge.

The partition coefficient of these heavy metals on the MP surface differed depending on the type of metal. Furthermore, heavy metal abundance in *Chironomus* sp. larvae was related to the heavy metal concentration of MPs isolated from wastewater sludge. This reveals that MPs can behave as heavy metal carriers, potentially polluting biota. The preceding section explained how colored (PES) MPs are easily swallowed by *Chironomus* species and how these polymeric chemical types have strong heavy metal adsorption capabilities (Selvam et al., 2021). The adsorption tendencies of Pb, Cd, Ni, and Cu on MP surfaces have been observed to be greater (Godoy et al., 2019). Heavy metals have been observed to be easily adsorbed on suspended particulate matter (SPM) due to their large surface area and reactivity (Liu et al., 2016). In the Bejan River system, the SPM partition coefficients of Cd, Cu, Pb, and Ni varied from 4.09 to 6.20. (Li et al., 2021). Heavy metals, on the other hand, were observed to have a strong tendency to adsorb on MPs with a larger partition coefficient. Heavy metals (Cd, Cu, Pb, and Ni, for example) have high MPS partition coefficient values in groundwater (Selvam et al., 2021; Sarkar et al., 2021). The present finding of a high MPs partition coefficient (Kps) supports the study (1.375–9). The concentration of the heavy metals was significantly higher (*p* < 0.001) in *Chironomus* larvae than in those detected in the sludge. This indicates the bioaccumulation of these metals inside the larvae. This result suggests that the ingestion of microfiber can be an additional way of causing heavy metal accumulation in *Chironomus* larvae (Zon et al., 2018). It can be concluded that once MPs reach the aquatic environment with their load of internal (additives) and external (environment) heavy metals, they can be ingested by lower aquatic organisms and may be transferred along the food chain until they reach humans (Carbery et al., 2018). According to previous studies (Harvey & Watts, 2018; Schwable et al., 2018), MPs have been found within human stools. These findings highlight that humans are exposed to MPs, and these particles can enter the human gut through the consumption of fish, bivalves, and crustaceans (Zakeri et al., 2020). This could result in the transport of not only MPs but also heavy metals to humans, affecting their immune systems and genetics (Brandts et al., 2018; Hwang et al., 2019).

Ecological factors that affect the adsorption of heavy metals on MPs still need future investigation.

## Conclusion

This is the first study to record MPs in upper Egypt wastewater systems. Our findings back up previous research that suggested *Chironomus* sp. could be useful as bioindicators for MPs in freshwater. Moreover, the use of *Chironomus* tubes as indicators for freshwater MPs is recorded for the first time in the current study. It can be an alarm for the accumulation of MPs in all compartments of the aquatic environment. Our results indicate that microfibers may impact aquatic fauna through their transport of different heavy metals. Based on this observation, further studies should be carried out on the bioaccumulation of MPs and associated contaminants (e.g., heavy metals, persistent organic compounds) within different higher trophic levels in different aquatic systems to realize the implications and risks of MPs as well as the toxicity resulting from the adsorbed presence of these contaminants in freshwater biota.

## Recommendations

- Preventive measures, such as the use of microfiber filters and innovative laundry detergents, may help reduce small MP emissions into wastewater.
- Furthermore, the establishment of methods for the management of discarded MPs, such as recycling products to restrict plastic input to different aquatic systems, is necessary.
- Finally, the concentration of MPs should be continuously monitored in the basin because wastewater is sometimes used to irrigate agricultural land.

## Data Availability

All data generated or analyzed during this study are included in this published article.
